# A single-stage dual-source inverter using low-power components and microcomputers

**DOI:** 10.1038/s41598-024-51513-w

**Published:** 2024-01-20

**Authors:** Majid Ghani Varzaneh, Amirhossein Rajaei, Navid Kamali-Omidi, Ali Shams-Panah, Mohamadreza Khosravi

**Affiliations:** 1 Shiraz University of Technology, Shiraz, Iran; 2 Islamic Azad University of Dezful, Dezful, Khozestan Iran; 3 Islamic Azad University of Najafabad, Najafabad, Isfahan, Iran; 4 Weifang University of Science and Technology, Shandong, China

**Keywords:** Electrical and electronic engineering, Power distribution

## Abstract

This paper is an attempt to provide a dual-source inverter, an intelligent inverter topology that links two isolated DC sources to a single three-phase output through single-stage conversion. The converter is designed to be utilized in hybrid photovoltaic fuel cell systems, among other renewable energy applications. The proposed dual-source inverter employs a single DC-AC converter, as opposed to conventional dual-source hybrid inverters which make use of several input DC-DC modules to obtain the voltage formed across the inverter’s input DC-link. In the proposed topology, the semiconductor count is low, which leads to improved efficiency, cost, complexity, and reliability. The proposed topology makes use of two impedance networks connected by transformers, diodes, and capacitors. The regulation of the electrical power generated by primary sources and the independence of the converter on key factors like voltage and frequency are essential parameters in multi-input converters. This feature becomes highly prominent when the control algorithm is implemented by conventional processors. Viewed from this perspective, the control method described in this paper is worthy of consideration. The research work describes a 220-W/50 Hz prototype that employs *Simple Boost-SPWM.* Experimental results verify the analyses and corroborate the satisfactory performance of the suggested converter.

## Introduction

Some renewable energy resources such as solar cells and wind turbines are widely available around the world and are good choices for energy storage systems. The intermittent nature of these energy sources requires them to be used alongside batteries or fuel cells. Energy generated by one of these renewable energy resources causes low reliability in supplying power to the grid system^1–3^. Photovoltaic panels and fuel cells generate not only low, but also unstable DC voltage. To overcome this shortcoming, power electronic boost converters should be used^1,2^. The terminal voltage could increase if additional photovoltaic cells are connected in series. Even using the second solution cannot compensate for the effect of partial shading^1,2^.

In hybrid energy systems with DC voltage sources, it is possible to use a separate DC-DC converter for each power source and then connect their outputs to a single DC bus. This solution, however, leads to higher costs, complexity, volume, and so on. The other proposed solution involves using multi-input converters^1,2,4,5^. These converters are powered by sources with different power and voltage levels, producing a fixed output of voltage and frequency^1,2,6,7^. In hybrid energy systems that take DC voltage as input and produce AC voltage as output, two technical approaches are available for conversion: single-stage and dual-stage^1^. Power electronic converters with a single or multiple input can be used to achieve these tasks. In dual-stage conversion structures, you need DC-DC converters. Some of these topologies are introduced in Refs.^3,4,6–8^. Somehow Multi-input converters must be used with single-stage conversion structures^1^. In this regard, some single-phase topologies are introduced in Refs.^9–13^, while three-phase topologies are discussed in Refs.^1,2,14–20^. Using a multi-input DC-AC converter for the implementation of single-stage energy conversion reduces the costs, weight, and volume^1^. Thus, the present research introduces a multi-input single-output DC-AC converter which belong to the category of single-stage conversion systems.

The topologies discussed in Refs.^17^^,^^18^ require three DC sources to generate a three-phase output. The topology discussed in Ref.^18^ needs three extra switches. Reference^19^ describes the development of a high-reliability multi-port inverter using five extra switches and no additional passive elements. The topologies proposed in Refs.^16,20,21^ are appropriate structures, especially for high-power applications. In this research study, the converters used are implemented using twelve additional switches. The impedance source inverter, initially covered in Ref.^22^, uses single-stage conversion by utilizing the "shoot-through" concept^23,24^. The Shoot-Through State (STS) in such structures increases the value of DC-link voltage. This new concept has recently been used to propose new designs for multi-input inverters^1,2,14,15,17^.

The topologies presented in Refs.^25–33^ are dual-stage multi-input inverters. These topologies are unique in several ways, including their low number of semiconductors and absence of low-frequency transformers. These features make them well-suited for photovoltaic and grid-connected applications. However, these systems do have some drawbacks, such as low voltage gain. Various types of inverters, such as multilevel, modified Z-source, and high step-up three-phase inverters, were discussed in Refs.^34–41^. These inverters have notable features such as being short-circuit risk-free, not requiring connection to the AC grid for stable output voltage, minimizing voltage and current stress, having higher voltage gain, continuous input current, and excellent power-sharing capabilities. The single-stage dual-input inverter design covered in Ref.^42^ carries a risk of short-circuit. Additionally, this inverter may need to be connected to the AC grid to maintain a consistent output voltage.

The new multi-port impedance source inverters introduced by Refs.^14^^,^^15^ form the basis of the z-source inverter presented in Ref.^22^. Reference^14^ describes a dual-input dual-output inverter with nine switches, allowing each source to supply a separate load. In the topology presented in Ref.^15^, the input sources cannot have random voltage or current levels. Two dual-input single-output three-phase inverters are discussed in Refs.^1,2^. In the topology developed by Ref.^2^, replacing the two inductors of the classic impedance source inverter with two transformers forms a new multi-port inverter. In this inverter, the DC-link voltage is a three-level signal with a specific switching frequency. Hence, a new modulation method is required to overcome the *THD* problem considering the classic modulation methods generating high *THD* at output. A multi-input inverter is proposed in Ref.^1^ using a z-source inverter^22^. This inverter uses a single-stage power conversion. The traditional z-source structure forms the basis of this topology^22^. This structure and the proposed topology use artificial intelligence for preventing negative bias of the capacitors placed in DC-Link bus. This topology has more components with respect to the proposed inverter in this paper.

The connections in the proposed inverter are such that when the DC-link capacitors (C_5_ and C_6_) start to charge up with a negative voltage, the diodes paralleled with these capacitors will start conducting. Therefore, the negative charge of the capacitors would be discharged into the other energy storage elements placed in *Z*1 through these diodes. So, this hardware-based approach will eliminate the need for excessive detection and protection mechanisms by humans or other additional hardware Infrastructure. This is a hardware-based artificial intelligence because human attention and protection do not play a role in the process. In this paper, "artificial intelligence" refers to machine intelligence. As it is known the machine intelligence is a sub-branch of artificial intelligence which implicates substituting human with machine hardware for taking human-based tasks and procedures. This approach uses high-capacity polarized electrolytic capacitors in the converter’s structure, reducing voltage ripple of the DC link section used in the inverters structure.

In this paper, a new single-stage multi-port inverter is proposed by removing the third windings of the transformers and some active and passive elements from the topology introduced in Ref.^1^. This inverter shows higher efficiency and an equal gain in real-life conditions. Improvements in cost, weight, volume, and other aspects have also been observed. The hardware-based artificial intelligence used in manufacturing DC-link capacitors does not receive negative voltage. However, other advantages of the topology presented in Ref.^1^ are observable in the suggested structure. This function is performed using microcomputers and low-power embedded systems.

In this topology, two impedance networks are linked through diodes, capacitors, and the winding of transformers, which mechanism has replaced inductors. As impedance networks and STS are utilized, the proposed inverter serves as a voltage booster. Due to using capacitors in series with the transformers, there are no voltage spikes over the semiconductors caused by the leakage inductances. Therefore, in this topology, using snubbers is not a necessity.

In this paper, there are two dependent and two independent variables. The DC-link voltage and the ratio of the power generated by the sources are considered dependent variables. One feature of the proposed inverter is that it allows individual control of the two dependent variables. Otherwise, each dependent variable is only controlled by one independent variable, and changes in one cannot bring about any changes in the other. Also, *Simple Boost-SPWM*, first presented in Ref.^22^, is used as the modulation method. Following the above introduction, section “Presenting and analyzing the proposed inverter” presents the operating modes of the suggested inverter and more. Section “An analysis of steady state relations and power-sharing” provides a more detailed analysis of power-sharing and mathematical relationships. Finally, the outcomes of the simulation and experiments are discussed and assessed in the concluding section “Reliability assessment”.

## Presenting and analyzing the proposed inverter

**Figure 1:**
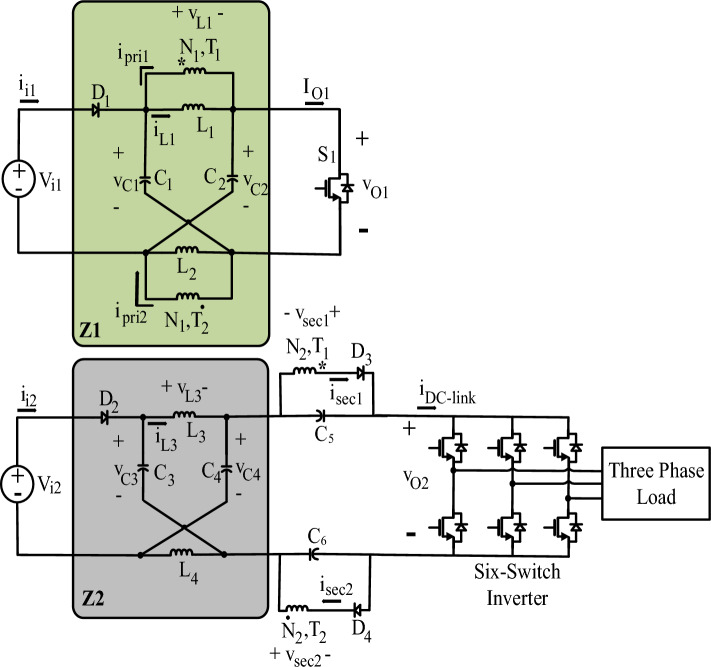
Proposed Dual-Source Three-Phase Inverter.

Figure 1 shows the inverter proposed in this paper. The inverter consists of two impedance networks (*Z*1 and *Z*2), two DC sources (*V*_*i*1_ and *V*_*i*2_), and a six-switch inverter. In *Z*1, two high-frequency transformers (*T*_1_ and *T*_2_) are used instead of two inductors, with their inductors (*L*_1_ and *L*_2_) taking the place of the removed inductors. *Z*1 enters the STS if *S*_1_ is turned on, and if *S*_1_ is turned off, *Z*1 enters the NSTS (Non-Shoot-Through State). If at least one leg of the six-switch inverter is shorted, *Z*2 enters the STS; otherwise, it enters the NSTS. The *V*_*i*1_ power is transferred to the load via *T*_1_, *T*_2_, *C*_5_, *C*_6_, and the six-switch inverter. The control of power generated by *V*_*i*1_ and *V*_*i*2_ is done by adjusting the shoot-through duty cycles of both *Z*1 and *Z*2.

Reference^22^ explains how *Z*1 works and discusses the relation between the average values of the voltages as follow:

1 $$\frac{\text{V}_{\text{C}1}}{\text{V}_{\text{i}1}} = \frac{1-\text{D}_{\text{st}1}}{1 - 2\text{D}_{\text{st}1}}$$

2 $$\frac{{\text{V}}_{{\text{O}}{1}}}{{\text{V}}_{{\text{i}}{1}}} = \frac{1}{{1}-{2}{\text{D}}_{{\text{st}}{1}}}$$

In (1) and (2), ${\text{D}}_{{\text{st}}{1}}$ is equal to the duty cycle of *S*_1_. Other variables are shown in Fig. 1. According to the circuit symmetry, it is possible to say that ${\text{V}}_{{\text{C}}{1}} = {\text{V}}_{{\text{C}}{2}}.$

**Figure 2:**
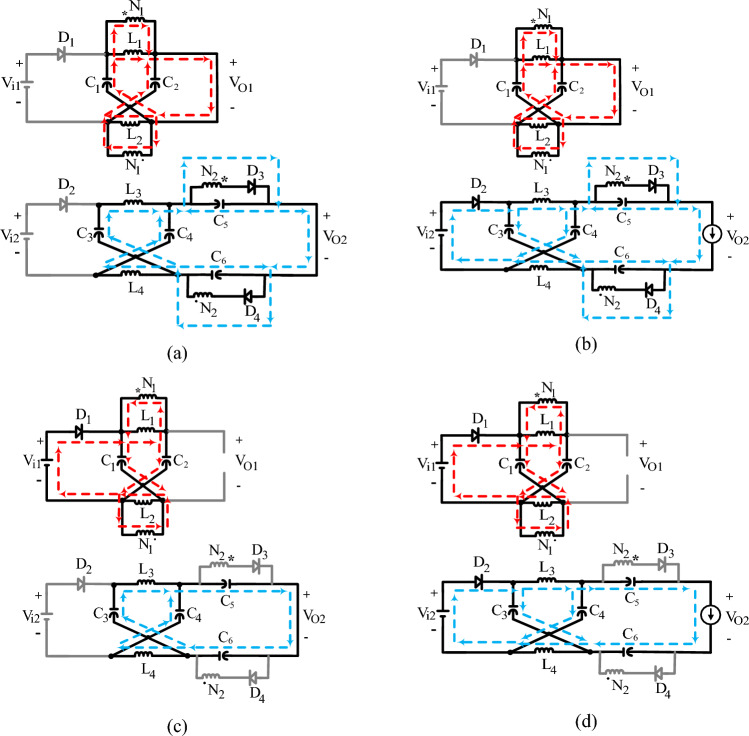
System circuits that are equivalent for (**a**) *Mode A*, (**b**) *Mode B*, (**c**) *Mode C*, and (**d**) *Mode D*. (The dashed red and blue lines, respectively, indicate *Z*1 and *Z*2 current paths.), (in *mode A* and *B*, *Z*1 is in STS and in *mode A* and *C*, *Z*2 is in STS).

Considering that all inductors of the inverter of Fig. 1 work in *CCM*, and as both *Z*1 and *Z*2 have two operation modes (STS and the NSTS), four operation modes can be defined for the proposed inverter: *Mode A, Mode B, Mode C, Mode D* as is shown in Fig. 2. Table 1 details each mode. What inductors or capacitors charge or discharge are displayed in Fig. 2. Once *Z*1 enters in the STS because *S*_1_ is a short circuit (according to Fig. 2a and b), the inductors of *Z*1, *C*_5_, and *C*_6_ are charged by the capacitors of *Z*1. When *Z*1 enters in the NSTS, the capacitors of *Z*1 are charged, and the inductors of *Z*1, *C*_5_, and *C*_6_ are discharged (according to Fig. 2c and d). In this case, the branch containing *S*_1_ is an open circuit.

**Table 1:** An explanation of semiconductors states in all modes.

Modes	When semiconductors are ON	When semiconductors are OFF
*A*	*D*_3_, *D*_4_, *S*_1_	*D*_1_*, D*_2_
*B*	*D*_2_, *D*_3_, *D*_4_, *S*_1_	*D*_1_
*C*	*D*_1_	*D*_2_, *D*_3_, *D*_4_, *S*_1_
*D*	*D*_1_*, D*_2_	*D*_3_, *D*_4_, *S*_1_

When *Z*2 enters in the STS (Cf. Fig. 2a and c), at least one of the legs of the six-switch inverter is a short circuit. At this time, similar to the *Z*1 operation, the capacitors of *Z*2 charge the inductors of *Z*2 and discharge themselves. When *Z*2 enters in the NSTS (according to Fig. 2b and d), the capacitors of *Z*2 are charged and the inductors of *Z*2 are discharged. At this time, the six-switch inverter connects the load to the other parts of the circuit, and the load current and the six-switch inverter's current become equal. The power is transferred from *V*_*i*1_ to the other parts of the circuit only if *Z*1 is in the NSTS, and the power is transferred from *V*_*i*2_ to the other parts of the circuit only if *Z*2 is in the NSTS.

In this topology, the components *C*_5_, *D*_3_ and secondary winding of *T*_1_ and also *C*_6_, *D*_4_ and secondary winding of *T*_2_ have the responsibility to transfer power from *V*_*i*1_ to the load. It is obvious that this portion of power is delivered to the load through an interconnection between the *Z*2 and load. Therefore, there is a need for a low ripple voltage in the *C*_5_ and *C*_6_ to reduce the load voltage and current ripple. This requires high-capacity capacitors, mainly found in the class of polarized electrolytic capacitors. So, it is possible to achieve this goal by using high-capacity electrolytic capacitors for *C*_5_ and *C*_6_.

Additionally, supposing that *Z*1 does not supply the *C*_5_ and *C*_6_, it can be said that *C*_5_ and *C*_6_, after some switching periods, will be charged with negative voltage by *Z*2. When this happens, the power will not be transferred to the load and the system will be inefficient.

Also, because *C*_5_ and *C*_6_ are polarized electrolytic capacitors, the negative voltage causes both an explosion and a lack of transfer of power to the load. Therefore, it can be said that the presence of *C*_5_ and *C*_6_ in DC-link, executed for power-sharing proposes, can cause a lack of supplying the load, if *Z*1 and the windings of *T*_1_ and *T*_2_ are unable to discharge the negative voltage of *C*_5_ and *C*_6_. One solution to overcome the above problem is using some sensing and relay circuits in parallel with *C*_5_ and *C*_6_. The solution requires several sensors, which increases the cost and foundation preparations. Also, the aging of the relays due to the moving elements requires to additional attentions. A better solution to the problem is using machine intelligence which is a sub-branch of artificial intelligence. Using machine intelligence for overcoming this problem can be performed by using controllers or hardware-based methods. The inverter proposed here makes use of machine intelligence as a hardware-based artificial intelligence so that if *C*_5_ and *C*_6_ are charged by a negative voltage, *Z*1, *D*_3_, *D*_4_, and the secondary windings of *T*_1_ and *T*_2_ will start to discharge them to *C*_1_, *C*_2_, *L*_1_, and *L*_2_. The energy stored in *C*_1_, *C*_2_, *L*_1_, and *L*_2_ in this way, thus, will be discharged to *C*_5_ and *C*_6_ to charge with positive voltage.

Furthermore, the capacitors *C*_5_ and *C*_6_ may be charged with negative voltage if ${\text{V}}_{{\text{i}}{1}} = {0}$. if this happens, the problem mentioned in the previous paragraphs will accrue. Therefore, in such cases also, some relay circuits or human monitoring are required to stop that from happening. The interesting point to note is that *Z*1 will cause discharge of *C*_5_ and *C*_6_, if they start to charge with a negative voltage. Also, machine intelligence is in operation in such situations.

The switching frequencies chosen for *Z*1 and *Z*2 in the suggested inverter might not be equal. This precludes the need for any additional consideration and the inverter works according to what was explained in this section. This research paper employs *Simple Boost-SPWM* introduced in Ref.^22^ as switching method for *Z*2.

## An analysis of steady state relations and power-sharing

For the suggested inverter illustrated in Fig. 1, if *Z*1 enters in the STS, *C*_5_ and *C*_6_ will be charged, and if Z1 enters in the NSTS, *C*_5_ and *C*_6_ will be discharged. Once *Z*1 enters in the STS, the voltage of *C*_1_ and *C*_2_ will drop across the primary windings of *T*_1_ and *T*_2_, respectively, multiplied by $\frac{{\text{N}}_{2}}{{\text{N}}_{1}}$ and dropped across *C*_5_ and *C*_6_. Considering these explanations and (1), it can be said that:

$$\frac{{\text{V}}_{{\text{C}}{5}}}{{\text{V}}_{{\text{C}}{1}}} = \frac{{\text{N}}_{2}}{{\text{N}}_{1}}= \text{n}$$

3 $$\frac{{\text{V}}_{{\text{C}}{5}}}{{\text{V}}_{{\text{i}}{1}}} = \frac{{1}-{\text{D}}_{{\text{st}}{1}}}{{1}-{2}{\text{D}}_{{\text{st}}{1}}}\text{.}\frac{{\text{N}}_{2}}{{\text{N}}_{1}}$$

According to the circuit symmetry, ${\text{V}}_{{\text{C}}{5}} = {\text{V}}_{{\text{C}}{6}}$ and ${\text{V}}_{{\text{C}}{3}} = {\text{V}}_{{\text{C}}{4}}$. Appling volt-second law for *L*_3_ yields:

4 $${\text{(V}}_{{\text{C}}{3}}\text{+}{2}{\text{V}}_{{\text{C}}{5}}\text{).}{\text{D}}_{{\text{st}}{2}}\text{+}\left({\text{V}}_{{\text{i}}{2}}-{\text{V}}_{{\text{C}}{3}}\right)\text{.}\left({1}-{\text{D}}_{{\text{st}}{2}}\right) = {0}$$

In (4), ${D}_{st2}$ is equal to the shoot-through duty cycle of *Z2.* Considering (1), (3), and (4), the following results:

5 $${\text{V}}_{{\text{C}}{3}}= \text{n} \left(\frac{{2}{\text{D}}_{{\text{st}}{2}}}{{1}-{2}{\text{D}}_{{\text{st}}{2}}}\right)\text{.}\left(\frac{{1}-{\text{D}}_{{\text{st}}{1}}}{{1}-{2}{\text{D}}_{{\text{st}}{1}}}\right)\text{.}{\text{V}}_{{\text{i}}{1}}\text{+}\left(\frac{{1}-{\text{D}}_{{\text{st}}{2}}}{{1}-{2}{\text{D}}_{{\text{st}}{2}}}\right)\text{.}{\text{V}}_{{\text{i}}{2}}$$

Using the same analysis, DC-link voltage (${\text{V}}_{{\text{O}}{2}}$)–when *Z*2 is in the NSTS – can yield as is shown in the following:

6 $${\text{V}}_{{\text{O}}{2}}\text{=n.}\left(\frac{{1}-{\text{D}}_{{\text{st}}{1}}}{{1}-{2}{\text{D}}_{{\text{st}}{1}}}\right)\left(\frac{2}{{1}-{2}{\text{D}}_{{\text{st}}{2}}}\right)\text{.}{\text{V}}_{{\text{i}}{1}}\text{+}\left(\frac{1}{{1}-{2}{\text{D}}_{{\text{st}}{2}}}\right)\text{.}{\text{V}}_{{\text{i}}{2}}$$

The suggested inverter is a voltage booster, as demonstrated by Eq. (6), making it a good fit for some such applications as hybrid photovoltaic-fuel cell systems. The suggested inverter's comparison with comparable structures presented in ^1,14,17^, from a voltage gain viewpoint, is performed in the following part.

**Figure 3:**
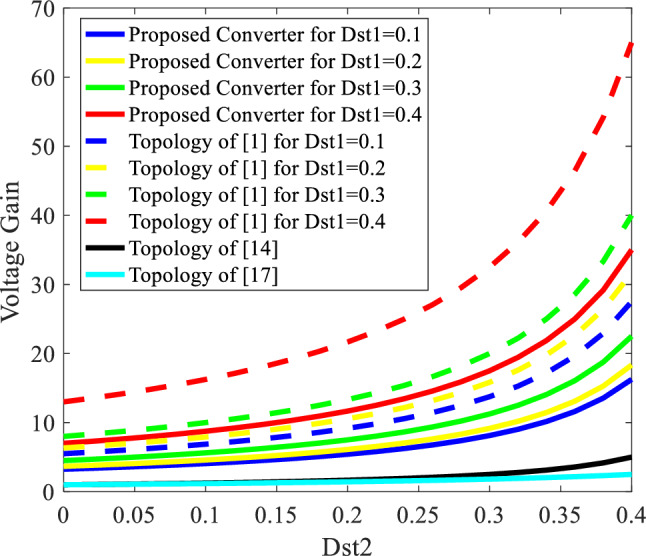
The voltage gain comparison between the topologies shown in Refs.^1,14,17^ and the suggested inverter.

To have a fair comparison, some such assumptions as ${\text{V}}_{{\text{i}}{1}} = {\text{V}}_{{\text{i}}{2}}$ for Refs.^1,14,17^, $\frac{{\text{N}}_{2}}{{\text{N}}_{1}} = {1}$ for the proposed converter, and $\frac{{\text{N}}_{3}}{{\text{N}}_{1}} = \frac{{\text{N}}_{2}}{{\text{N}}_{1}} = {1}$ for Ref.^1^ are taken into account. The results are illustrated in Fig. 3. According to the results displayed in Fig. 3, it can be stated that the voltage gain of the proposed inverter is higher than the topologies of Refs.^14,17^ for all values of ${\text{D}}_{{\text{st}}{1}}$. But the topology of Ref.^1^ shows a higher gain than the proposed inverter in the same value of ${\text{D}}_{{\text{st}}{1}}$ and ${\text{D}}_{{\text{st}}{2}}$. For instance, when ${\text{D}}_{{\text{st}}{1}} = {\text{D}}_{{\text{st}}{2}} = {0.2}$, the voltage gain of the suggested inverter is 9.9, 6.11, 1.67, and 1.43, according to the topology of Refs,^1,14^^,^^17^. The gain of the suggested inverter will be greater than that of the inverters suggested in Refs.^14,17^ and will be lower than the inverter suggested in Ref.^1^ due to the increase in transformer turns ratio. This comparison is performed under ideal conditions where parasitic elements of the converters are not taken into account and the efficiency of all converters is assumed to be %100. As will be explained in Part IV, in real-life conditions, where parasitic elements of topologies are considered, the efficiency of the proposed inverter is higher than the topology of Ref.^1^. Also, the voltage gain of the proposed inverter is almost equal to that of the topology of Ref.^1^ in high-power applications.

The act of controlling the power produced by each source is crucial for multi-input converters^1^. In this paper, the role of each source in providing the load is represented by the index $\frac{{\text{P}}_{{\text{i}}{1}}}{{\text{P}}_{{\text{i}}{2}}}$. Furthermore, ${\text{V}}_{{\text{O}}{2}}$ is an additional index that has to be under control. In the suggested system, the independent variables are ${\text{D}}_{{\text{st}}{1}}$ and ${\text{D}}_{{\text{st}}{2}}$. Therefore, there are two dependent variables (${\text{V}}_{{\text{O}}{2}}$ and $\frac{{\text{P}}_{{\text{i}}{1}}}{{\text{P}}_{{\text{i}}{2}}}$) that have to be controlled by ${\text{D}}_{{\text{st}}{1}}$ and ${\text{D}}_{{\text{st}}{2}}$. Equation (6) depicts the relation between ${\text{V}}_{{\text{O}}{2}}$, ${\text{D}}_{{\text{st}}{1}}$, and ${\text{D}}_{{\text{st}}{2}}$. So, another variable is required to show the relation between $\frac{{\text{P}}_{{\text{i}}{1}}}{{\text{P}}_{{\text{i}}{2}}}$,${\text{D}}_{{\text{st}}{1}}$, and ${\text{D}}_{{\text{st}}{2}}$. When *Z*2 is in the STS, it can be said that:

7 $${\text{I}}_{\text{DC-link}}^{\text{st}} = {2}{\text{I}}_{{\text{L}}{3}}$$

where the average value of ${\text{i}}_{\text{DC-link}}$ during shoot-through time is represented by ${\text{I}}_{\text{DC-link}}^{\text{st}}$. Again, when *Z*2 is in the NSTS, this can be stated as:

8 $${\text{I}}_{\rm{DC-link}}^{\text{non st}}\rm{=}{\text{I}}_{\rm{Load}}$$

where the average values of ${\text{i}}_{\text{DC-link}}$ and load current in non-shoot-through time are denoted by ${\text{I}}_{\text{DC-link}}^{\text{non st}}$ and ${\text{I}}_{\text{Load}}$, respectively. Simultaneously taking into account (7) and (8), the average value of ${\text{i}}_{\text{DC-link}}$ in a single switching period can be obtained as Eq. (9).

9 $${\text{I}}_{\rm{DC-link}} = {2}{\rm{I}}_{{\text{L}}{3}}\rm{.}{\text{D}}_{{\rm{st}}{2}}\text{+}{\rm{I}}_{\text{Load}}\text{.}\left({1}-{\rm{D}}_{{\text{st}}{2}}\right)$$

As regards ${\text{I}}_{\rm{DC-link}} = {\rm{I}}_{{\text{L}}{3}}$ and ${\text{I}}_{{\text{L}}{3}} = {\text{I}}_{{\text{i}}{2}}$, (9) can be simplified as follow:

10 $${{\text{I}}_{{\text{i}}{2}}\text{.}\left({1}-{{2}{\text{D}}}_{{\text{st}}{2}}\right)= \text{I} }_{\text{Load}}\text{.}\left({1}-{\text{D}}_{{\text{st}}{2}}\right)$$

Therefore, the load's absorption of active power is:

11 $${\text{P}}_{\rm{o}} = {\text{V}}_{{\rm{O}}{2}}\text{. }{\rm{I}}_{\text{Load}}\rm{.}\left({1}-{\text{D}}_{{\rm{st}}{2}}\right)$$

The active power generated by source 2 is:

12 $${\text{P}}_{{\rm{i}}{2}} = {\rm{V}}_{{\text{i}}{2}}\rm{.}{\text{I}}_{{\rm{i}}{2}}$$

Considering (10), (11), and (12) simultaneously yields:

13 $${\text{P}}_{{\rm{i}}{2}} = \frac{{\rm{P}}_{\text{o}}\rm{.}{\text{V}}_{{\rm{i}}{2}}}{{\text{V}}_{{\rm{o}}{2}}\text{.}\left({1}-{{2}{\rm{D}}}_{{\text{st}}{2}}\right)}$$

Also, the generated active power by source 1 is:

14 $${\text{P}}_{{\text{i}}{1}} = {\text{P}}_{\text{o}}-{\text{P}}_{{\text{i}}{2}}$$

Substituting (13) in (14) simplifies (14) as follow:

15 $${\text{P}}_{{\text{i}}{1}} = {\text{P}}_{\text{o}}\text{.}\left({1}-\frac{{\text{V}}_{{\text{i}}{2}}}{{\text{V}}_{{\text{o}}{2}}\text{.}\left(\text{1} - {{2}{\text{D}}}_{{\text{st}}{2}}\right)}\right)$$

The division of (15) by (13) yields the generated power ratio by the sources as follows:

16 $$\frac{{\text{P}}_{{\text{i}}{1}}}{{\text{P}}_{{\text{i}}{2}}} = \frac{{\text{V}}_{{\text{o}}{2}}\text{.}\left({1}-{{2}{\text{D}}}_{{\text{st}}{2}}\right)-{\text{V}}_{{\text{i}}{2}}}{{\text{V}}_{{\text{i}}{2}}}$$

Substituting (6) in (16) results:

17 $$\frac{{\text{P}}_{{\text{i}}{1}}}{{\text{P}}_{{\text{i}}{2}}} = \frac{{2}\text{n.}\left(\frac{{1}-{\text{D}}_{{\text{st}}{1}}}{{1}-{2}{\text{D}}_{{\text{st}}{1}}}\right)\text{.}{\text{V}}_{{\text{i}}{1}}}{{\text{V}}_{{\text{i}}{2}}}$$

Equation (17) shows that the control of $\frac{{\text{P}}_{{\text{i}}{1}}}{{\text{P}}_{{\text{i}}{2}}}$ is performed by adjusting ${\text{D}}_{{\text{st}}{1}}$, and whatever changes in ${\text{D}}_{{\text{st}}{2}}$ cannot bring about any changes in $\frac{{\text{P}}_{{\text{i}}{1}}}{{\text{P}}_{{\text{i}}{2}}}$. The reason is that in a real system, the control of the variable is performed by controllers, and the controller needs programming. Hence, simple control relations mean both easier implementation and faster execution. With these explanations, one can say that, as (17) only depends on ${\text{D}}_{{\text{st}}{1}}$, implementing (17) in the controller proves simple and the execution is affected in a speedy manner. By replacing (17) in (6), the following is obtained:

18 $${\text{V}}_{{\text{O}}{2}} = \left(\frac{{\text{P}}_{{\text{i}}{1}}}{{\text{P}}_{{\text{i}}{2}}}\right)\text{.}\left(\frac{1}{{1}-{2}{\text{D}}_{{\text{st}}{2}}}\right)\text{.}{\text{V}}_{{\text{i}}{2}}$$

Equation (18) well indicates that controlling ${\text{V}}_{{\text{O}}{2}}$ only depends on ${\text{D}}_{{\text{st}}{2}}$ and is independent of ${\text{D}}_{{\text{st}}{1}}$ if $\frac{{\text{P}}_{{\text{i}}{1}}}{{\text{P}}_{{\text{i}}{2}}}$ is already determined. Thus, by changing the values of ${\text{D}}_{{\text{st}}{1}}$ and ${\text{D}}_{{\text{st}}{2}}$, respectively, the values of $\frac{{\text{P}}_{{\text{i}}{1}}}{{\text{P}}_{{\text{i}}{2}}}$ and ${\text{V}}_{{\text{O}}{2}}$ can both be controlled. Correspondingly, (17) and (18) determine ${\text{D}}_{{\text{st}}{1}}$ and ${\text{D}}_{{\text{st}}{2}}$.

**Figure 4:**
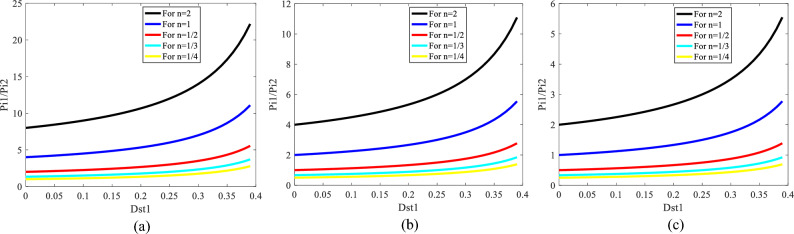
Curves of $\frac{{\text{P}}_{{\text{i}}{1}}}{{\text{P}}_{{\text{i}}{2}}}$ with respect to ${\text{D}}_{{\text{st}}{1}}$ for different values of ${\text{V}}_{{\text{i}}{1}}$, ${\text{V}}_{{\text{i}}{2}}$, and $n$. (**a**) for $\frac{{\text{V}}_{{\text{i}}{1}}}{{\text{V}}_{{\text{i}}{2}}} = {2}$, (**b**) for $\frac{{\text{V}}_{{\text{i}}{1}}}{{\text{V}}_{{\text{i}}{2}}} = {1}$, (**c**) for $\frac{{\text{V}}_{{\text{i}}{1}}}{{\text{V}}_{{\text{i}}{2}}} = {1/2}$.

Considering (17), the curves of $\frac{{\text{P}}_{{\text{i}}{1}}}{{\text{P}}_{{\text{i}}{2}}}$ are drawn in terms of different values of ${\text{V}}_{{\text{i}}{1}}$, ${\text{V}}_{{\text{i}}{2}}$, and $n$ illustrated in Fig. 4. This figure clearly shows that in the proposed inverter, regardless of the parameter values, the controller can manage $\frac{{\text{P}}_{{\text{i}}{1}}}{{\text{P}}_{{\text{i}}{2}}}$ in a wider range of cases by changing ${\text{D}}_{{\text{st}}{1}}$.

In order to compare the voltage and current stresses of the MOSFETs used in this paper with those in reference ^1^, the relevant relations are listed in Table 2.

**Table 2:** Comparing the voltage and current stresses of s2 to s7 in the proposed inverter with those in the inverter presented in Ref.^1^.

	Proposed inverter	Inverter presented in Ref.^1^
S_1_ current stress	$\frac{{\text{I}}_{{\text{i}}_{1}}}{{1}-{\text{D}}_{{\text{st}}{1}}}$	$\frac{{\text{I}}_{{\text{i}}_{1}}}{{1}-{\text{D}}_{{\text{st}}{1}}}$
S_2_ to S_7_ current stresses	${2}\text{.}{\text{I}}_{{\text{L}}_{3}\text{,avg}} = \frac{{\text{I}}_{{\text{i}}_{2}}}{{1}-{\text{D}}_{{\text{st}}{2}}}$	${2}\text{.}{\text{I}}_{{\text{L}}_{3}\text{,avg}} = \frac{{\text{I}}_{{\text{i}}_{2}}}{{1}-{\text{D}}_{{\text{st}}{2}}}$
S_1_ voltage stress	${\text{V}}_{{\text{O}}_{1}} = \frac{{\text{V}}_{{\text{i}}{1}}}{{1}-{2}{\text{D}}_{{\text{st}}{1}}}$	${\text{V}}_{{\text{O}}_{1}} = \frac{{\text{V}}_{{\text{i}}{1}}}{{1}-{2}{\text{D}}_{{\text{st}}{1}}}$
S_2_ to S_7_ voltage stresses	${\text{V}}_{{\text{O}}_{2}}\text{=n.}\left(\frac{{1}-{\text{D}}_{{\text{st}}{1}}}{{1}-{2}{\text{D}}_{{\text{st}}{1}}}\right)\left(\frac{2}{{1}-{2}{\text{D}}_{{\text{st}}{2}}}\right)\text{.}{\text{V}}_{{\text{i}}{1}}\text{+}\left(\frac{1}{{1}-{2}{\text{D}}_{{\text{st}}{2}}}\right)\text{.}{\text{V}}_{{\text{i}}{2}}$	${\text{V}}_{{\text{O}}_{2}} = {2}\text{.}\left(\frac{1}{{1}-{2}{\text{D}}_{{\text{st}}{2}}}\right)\text{.}\left(\frac{{\text{N}}_{2}}{{\text{N}}_{1}}\text{.}\frac{{1}-{\text{D}}_{{\text{st}}{1}}}{{1}-{2}{\text{D}}_{{\text{st}}{1}}}\text{+}\frac{{\text{N}}_{3}}{{\text{N}}_{1}}\text{.}\frac{{\text{D}}_{{\text{st}}{1}}}{{1}-{2}{\text{D}}_{{\text{st}}{1}}}\right)\text{.}{\text{V}}_{{\text{i}}{1}}\text{+}\left(\frac{1}{{1}-{2}{\text{D}}_{{\text{st}}{2}}}\right)\text{.}{\text{V}}_{{\text{i}}{2}}$

As shown in Table 2, the current and voltage stress of *S*_*1*_ and the current stress of *S*_*2*_ to *S*_*7*_ are parametrically the same in both cases. However, the voltage stress of *S*_*2*_ to *S*_*7*_ in both cases are not equal. The voltage stress of these switches is coupled to the transformers turn ratio with the definition of *n* $= \frac{{\text{N}}_{3}}{{\text{N}}_{1}} = \frac{{\text{N}}_{2}}{{\text{N}}_{1}}$. Therefore, in order to have a fairly comparison, the value of turns ratio is assumed to be unity. After applying this assumption and doing simplification for the voltage stress terms, it can be inferred that the voltage stress of *S*_2_ to *S*_7_ is smaller than the voltage stress of *S*_2_ to *S*_7_ in the topology presented in Ref.^1^ by a factor of $({1}-{\text{D}}_{{\text{st}}{1}})$.

## Reliability assessment

This section provided an assessment of the proposed system, focusing on estimating the reliability of the semiconductor switches, such as MOSFETs and diodes and so the whole converter. As previously mentioned, the system's machine intelligence feature ensures that not all semiconductor switches equally impact system operations. Specifically, the system's operation is critically dependent only on H-bridge MOSFETs (*S*_2_ to *S*_7_), as the system's operation and power transfer from *V*_*i*2_ to the load is maintained in the failure occurrence for other semiconductor switches. The power transmission from *V*_*i*2_ to the load becomes faulty when switches *S*_2_ to *S*_7_ fail. Therefore, only switches *S*_2_ to *S*_7_ affect the reliability estimation of the system. According to references^43,44^, the reliability of the H-bridge switches can be calculated using the following equation:

19 $${\text{R}}\left({\text{t}}\right)\text{ } = {\text{ e}}^{-{\lambda }_{t} \, \times \, {\text{t}}}$$

where ${\text{R}}\left({\text{t}}\right)$ is the reliability of the entire system, ${\lambda }_{\text{t}}$ is the total failure rate of the MOSFETs used in the H-bridge, and ${\text{t}}$ is the period over which the reliability is calculated. This paper evaluates the reliability of H-bridge MOSFETs by calculating the failure rate using the modern prediction standard IEC-TR-62380^45–48^. According to the IEC-TR-62380 standard, a mission profile is needed to calculate the failure rate based on the system's operating and environmental conditions. The mission profile table contains various parameters necessary for calculating the reliability of a system or component, taking into account all the conditions encountered during its operational lifespan. This includes environmental conditions, usage, and other factors that can impact the performance and reliability of the system or component. Table 3 presents the mission profile well-suited for the system studied in this paper. As can be seen in this table, ${\left({\text{t}}_{\text{ae}}\right)}_{\text{i}}$ is the average outside ambient temperature surrounding the equipment, during the *ith* phase of the mission profile, ${\left({\text{t}}_{\text{ac}}\right)}_{\text{i}}$ represents the average ambient temperature of the printed circuit board (PCB) near the components, where the temperature gradient is cancelled, ${\tau }_{\text{i}}$ is the *ith* working time ratio of the transistor for the *ith* junction temperature of the mission profile, ${\tau }_{\text{on}}$ is the total working time ratio of the transistor, ${\tau }_{\text{off}}$ is the time ratio for the transistor being in storage (or dormant) mode, and ${\text{n}}_{\text{i}}$ is the Annual number of cycles.

**Table 3:** Definition of parameters for mission profile^45^.

Environment type	Equipment type	${\left({\text{t}}_{\text{ae}}\right)}_{\text{i}}$	${\left({\text{t}}_{\text{ac}}\right)}_{\text{i}}$	${\tau }_{\text{i}}$	${\tau }_{\text{on}}$	${\tau }_{\text{off}}$	${\text{n}}_{\text{i}}$ $\text{cycles/year}$
Ground benign: (G_B_)	Switching	20	30	1	1	0	365

After determining the appropriate mission profile, it is important to calculate the total power loss for each MOSFET in order to estimate the junction temperature of each switch which is used in failure rate calculation. The total power loss for each MOSFET includes switching loss and conduction loss, which need to be calculated first^47^. The factors required for estimating power loss of MOSFETs are illustrated in Table 4. As can be seen in this table, the values of *t*_*rise*_, *t*_*fall*_ and *R*_*on*_ are respectively the rise time, fall time and the on-state resistance of the MOSFETs which are extracted from the datasheet. Additionally, *I*_*switch,max*_, *I*_*switch,rms*_ and ${\text{V}}_{\text{DS,applied}}$ are respectively the maximum value of drain current, RMS value of the drain current and the maximum value of drain-source voltage impinged to the MOSFET. Also, *FF* and *F*_*sw*_ are the fundamental frequency of the load current and the switching frequency of the MOSFETs respectively.

**Table 4:** Factors required for calculating power loss^49^.

Parameter	*t*_*rise*_ (*ns*)	*t*_*fall*_ (*ns*)	*R*_*on*_ (*Ω*)	*I*_*switch,max*_ (*A*)	*I*_*switch,rms*_ (*A*)	${\text{V}}_{\text{DS,applied}}$(*V*)	*FF* (*Hz*)	*F*_*sw*_ (*KHz*)
Value	59	58	0.27	3.7	1.47	94	50	4

Therefore, the total power loss for each MOSFET is calculated by Eq. (20) as follows:

20 $${\text{P}}_{\text{Loss-MOSFET}}\text{ = }{\text{P}}_{\text{sw}}\text{ } + {\text{ P}}_{\text{cond}}$$

In (20), ${\text{P}}_{\text{Loss-MOSFET}}$ is the total power loss for each MOSFET, ${\text{P}}_{\text{sw}}$ represents the switching loss for each MOSFET, and ${\text{P}}_{\text{cond}}$ is the conduction loss for each MOSFET. The conduction loss of the MOSFET is calculated by Eq. (21) as follows:

21 $${\text{P}}_{\text{cond}} =  0.5 \times {\text{R}}{\text{on}}\times {I}_{switch, rms}^{2}$$

Therefore, by substituting the required values from Table 4, the conduction loss for each MOSFET is equal to ${\text{P}}_{\text{cond}}\; = 0.583 \text{W}$. Since the load absorbs a sinusoidal current from the H-Bridge, because a Three-Phase filter with a constant fundamental frequency of 50 Hz was utilizes, any change in the load current will only affect the peak value of the load current. So, A correction factor called *K* is needed to calculate the average of the maximum variable value of the drain current. Therefore, the switching loss for each MOSFET is calculated using the following equation^47,48^.

22 $${\text{P}}_{\text{sw}} = 0.5{\text{V}}_{\text{DS,applied }}\text{.}{\text{I}}_{\text{switch,max}}\text{ . K . (t}\text{rise} + {\text{t}}\text{fall)}\text{ . FF}$$

where the correction factor is calculated as $\text{K} = \sum_{\rm{n} = {1}}^{\text{Q}}{\rm{sin}}\left(\frac{{2}\pi }{\text{Q}}{\rm{n}}\right)= 50.9$ in which *Q* is called frequency ratio in which can be calculated as $\text{Q= }\frac{{\text{f}}_{\text{sw}}}{\text{FF}} = 80$. By substituting the value of correction factor and other essential factors from Table 4 in (22), the switching loss value would be equal to ${\text{P}}_{\text{sw}} = 0.052 \text{W}$. Therefore, the total power loss for each MOSFET is equal to ${\text{P}}_{\text{Loss-MOSFET}} = 0.635 \text{W}$.

Based on IEC-TR-62380 standard, the general formula for calculating the MOSFET failure rate can be expressed as follows:

23 $${\lambda }_{\text{MOSFET}} = \left(\left\{\left({\lambda }_{0}{\pi }_{\text{S}}\right)\text{.}\left(\frac{\sum {{\text{(}\pi }_{\text{t}}\text{)}}_{\text{i}} \, {\tau }_{\text{i}}}{{\tau }_{\text{on}}\text{+}{ \, \tau }_{\text{off}}}\right)\right\}+\left\{\left(2.75\times {10}^{-3}\sum {{\text{(}\pi }_{\text{n}}\text{)}}_{\text{i}}{{\text{(}\Delta {\text{T}}}_{\text{i}}\text{)}}^{0.68}\right)\text{.}{\lambda }_{\text{B}}\right\}+\left\{{\text{(}\pi }_{\text{I}}\text{.}{ \, \lambda }_{\text{EOS}}\right\}\right)\times {\text{(}{10}}^{-9}\text{failure/year}\text{)}\text{ or} FIT$$

In Eq. (23), ${\pi }_{\text{S}}$ is charge factor, ${\lambda }_{0}$ represents the base failure rate of the MOSFET, ${{\text{(}\pi }_{\text{t}}\text{)}}_{\text{i}}$ is the *i*th temperature factor related to the *i*th junction temperature of the MOSFET mission profile, ${{\text{(}\pi }_{\text{n}}\text{)}}_{\text{i}}$ is the *i*th influence factor related to the annual cycles of thermal variations experienced by the MOSFETs package, ${\Delta {\text{T}}}_{\text{i}}$ is the *i*th thermal amplitude variation of the mission profile, ${\lambda }_{\text{B}}$ is base failure rate of the MOSFET package, ${\pi }_{\text{I}}$ is the influence factor related to the use of the MOSFETs body diode, and ${\lambda }_{\text{EOS}}$ is failure rate related to the electrical overstress in the considered application. Table 5 shows the mathematical expressions and parameter values used in Eq. (23).

**Table 5:** Calculated factors used in failure rate formula^45^.

Factor	Mathematical expression	value	Description	
${\lambda }_{0}$	*–*	2 (*FIT*)	for MOSFET based on IEC-TR-62380	
*S*_1_	$\frac{{\text{V}}_{\text{DS,applied }}}{{\text{V}}_{\text{DS,max}}}$	0.188	Based on the datasheet and operating conditions:	$$\left\{\begin{array}{c}{\text{V}}_{\text{DS,applied }} = \, {94} \, {\text{V}}\\ {\text{V}}_{\text{DS,max}} = \, {500} \, {\text{V}}\end{array}\right.$$
*S*_2_	$\frac{{\text{V}}_{\text{GS,applied }}}{{\text{V}}_{\text{GS,max}}}$	1	Based on the datasheet and operating conditions:	$$\left\{\begin{array}{c}{\text{V}}_{\text{GS,applied }}={20} {\text{V}}\\ {\text{V}}_{\text{GS,max}}={20} {\text{V}}\end{array}\right.$$
${\pi }_{\text{S}}$	$\text{0.22}{\text{e}}^{\text{1.7}{\text{S}}_{1}} \, \times \, \text{0.22}{\text{e}}^{{3}{\text{S}}_{2}}$	1.34	*–*	
${\tau }_{\text{i}}$	*–*	1	Based on mission profile	
${\tau }_{\text{on}}$	*∑* ${\tau }_{\text{i}}$	1	Based on mission profile	
${\tau }_{\text{off}}$	*–*	0	Based on mission profile	
${\text{n}}_{\text{i}}$	*–*	365 (*cycle/year*)	Based on mission profile	
${{\text{(}\pi }_{\text{n}}\text{)}}_{\text{i}}$	${{\text{n}}_{\text{i}}}^{0.76}$	88.58	Based on mission profile ${\text{n}}_{\text{i }}= \text{ } {365}$	
${\Delta {\text{T}}}_{\text{i}}$	$\frac{{\Delta {\text{T}}}_{\text{j}}}{3}\text{ } + { \left({\text{t}}_{\text{ac}}\right)}_{\text{i}} \text{- }{\left({\text{t}}_{\text{ae}}\right)}_{\text{i}}$	$\text{18.46}^\circ {\text{C}}$	For junction temperature range: ${\Delta {\text{T}}}_{\text{j}}\text{ } = {\text{ RTH}}_{\text{ja}}\times {\text{ P}}_{\text{Loss-MOSFET}}\text{ = }{40}\,^\circ \text{C/W} \, \times \, \text{0.635W}\text{ = }\text{25.4}\,^\circ {\text{C}}$	
${{\text{(}\pi }_{\text{t}}\text{)}}_{\text{i}}$	${\text{e}}^{{3480}\left(\frac{1}{{373}} \text{- }\frac{1}{{\text{T}}_{\text{j }}+ \text{ 273} }\right)}$	0.28	For junction temperature: ${\text{T}}_{\text{j }} = { \, \Delta {\text{T}}}_{\text{j}} \, + \, {\left({\text{t}}_{\text{ac}}\right)}_{\text{i}}= \text{ } \text{25.4} \, + \, {30}\text{ = }\text{55.4}\,^\circ {\text{C}}$	
${\lambda }_{\text{B}}$	*–*	6.9 (*FIT*)	Based on IEC-TR-62380 for TO-247 package	
${\pi }_{\text{I}}$	*–*	0	Based on IEC-TR-62380 for non-interface	
${\lambda }_{\text{EOS}}$	*–*	40 (*FIT*)	Based on IEC-TR-62380 for non-interface	

**Figure 5:**
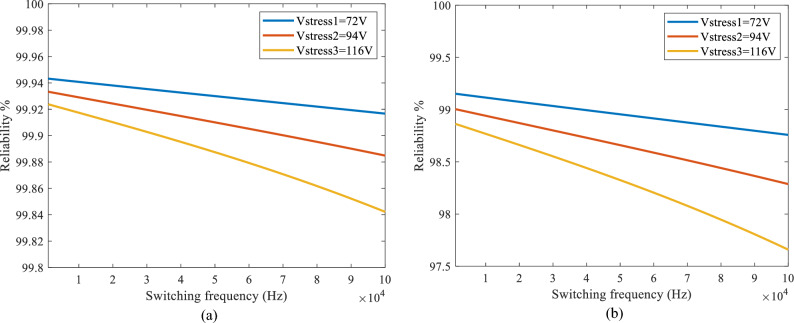
The calculated reliability curves for different voltages and frequencies applied to *S*_2_ to *S*_7_ (**a**) 1 year period (**b**) 15 years period.

Ultimately, substituting the required values from Table 5 into Eq. (23) the failure rate for a single MOSFET in the proposed inverter is calculated as ${\lambda }_{\text{MOSFET}} = 12.95 \text{ (FIT)}$. So, the failure rate for six MOSFETs used in H-bridge structure will be equal to ${\lambda }_{\text{6-MOSFET}}\text{ } = { \, {6}\times \lambda }_{\text{MOSFET}} = \text{77.74 (FIT)}$. Eventually by substituting the ${\lambda }_{\text{6-MOSFET}}$ in (19) for operating period of one year $\left(\text{t } = {365}\times {24}\right)$ the reliability of the system would be equal to ${\text{R}}\left({\text{t}}\right) = {99.93 \%}$. As it is known switches such as MOSFETs are very sensitive to the increase in their operating voltage and temperature. In these conditions, voltage and thermal accelerations cause aging chemical reactions to speed up the failure process and increase the failure rate of the MOSFET. Also, as it is known power electronic converters are designed for long life spans in order to be efficient, cost effective and to have acceptable performance in this period. Therefore, the reliability of the proposed inverter was calculated for 1 and 15 years of operation considering three different voltage stresses of 72, 94,116 and a switching frequency range of 1KHZ to 100KHZ for the MOSFETs. In this procedure different voltage stresses and switching frequencies were chosen in order to analyze their impact on the overall system's reliability. The Fig. 5a and b show how the reliability varies at different frequencies for three different voltage stresses for the MOSFETs *S*2 to *S*7 at two different time periods.

As can be seen in Fig. 5a the reliability of the system for all voltage stresses and switching frequencies is above 99.8% which is a good number that ensures a well performance for the system in aone-year period. Also, as can be seen in Fig. 5b the reliability of the system for all voltage stresses and switching frequencies is above 97.5% which is also a fairly good number that ensures the well performance for the system in the period of fifteen-years.

## Simulation and experimental results

**Figure 6:**
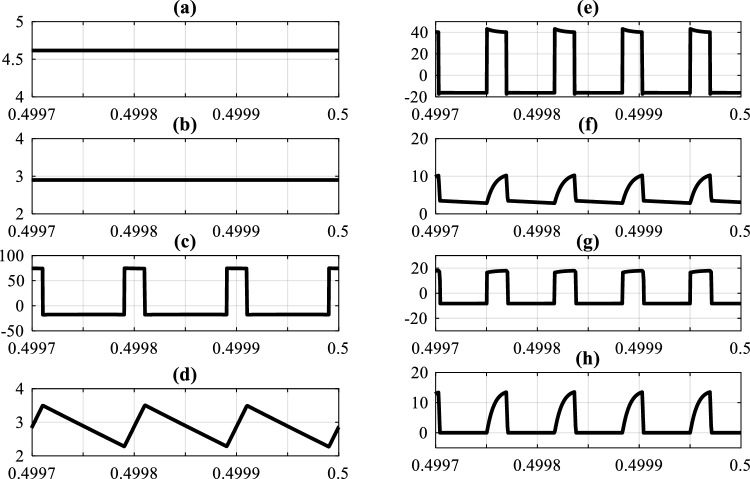
Simulation results: (**a**) *I*_*i*1_, (**b**) *I*_*i*2_, (**c**) *v*_*L*3_ or *v*_*L*4_, (**d**) *i*_*L*3_ or *i*_*L*4_, (**e**) *v*_*L*1_ or *v*_*L*2_, (**f**) primary current of transformers (*i*_*pri*1+_ *i*_*L*1_ or *i*_*pri*2+_ *i*_*L*2_), (g) *v*_*sec*1_ or *v*_*sec*2_, (h) *i*_*sec*1_ or *i*_*sec*2_. (Horizontal axes are in seconds).

**Figure 7:**
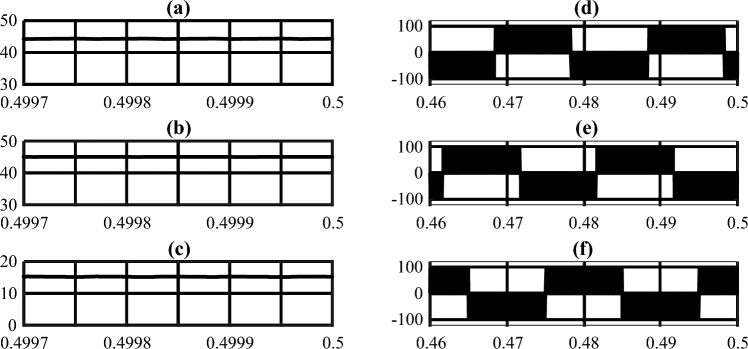
Simulation results: (**a**) *V*_*C*1_ or *V*_*C*2_, (**b**) *V*_*C*3_ or *V*_*C*4_, (**c**) *V*_*C*5_ or *V*_*C*6_, (**d**–**f**) Line to Line voltages (Horizontal axes are in seconds).

**Figure 8:**
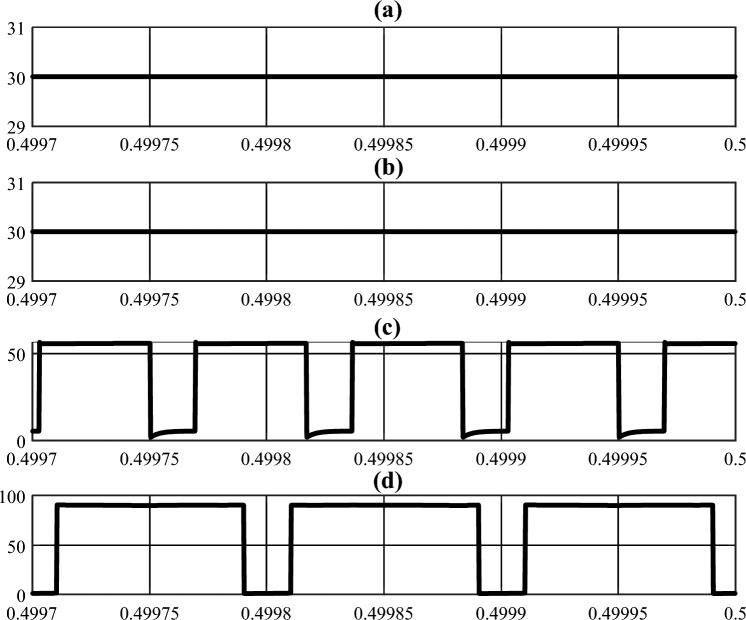
Simulation results: (**a**) *V*_*i*1_, (**b**) *V*_*i*2_, (**c**) *V*_*O*1_, (**d**) *V*_*O*2_ (Horizontal axes are in seconds).

**Figure 9:**
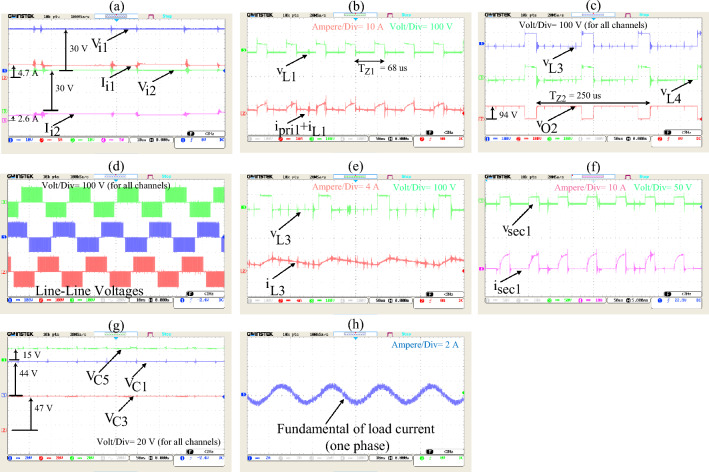
Experimental results (Time/div: for (**a**) is 10 $\mu$ s, for (**b**,**c**,**e**–**g**) is 50 $\mu$ s, for (**d**) and (**h**) is 10 ms.), (${\text{T}}_{{\text{Z}}{1}}$ and ${\text{T}}_{{\text{Z}}{2}}$ are switching period of *Z*1 and *Z*2, respectively), (Volt/div and Ampere/div for each carve are added in each part with specific colors).

A 220-W prototype was built and tested to assess the performance of the suggested inverter in actual operating circumstances. In the tests, the input sources were connected to the proposed inverter via LC filters so that the input currents were placed nearer to the DC component. The system parameters are shown in Table 6. As can be seen in this table, the duty cycle is set to a low value in order to reduce both the input current harmonics and the load voltage harmonics. that brings the prominent advantage of producing high voltages in low duty cycle conditions for the proposed system. To demonstrate and validate the suggested inverter's capability to operate at two distinct frequencies simultaneously for *Z*1 and *Z*2, the switching frequencies for *Z*1 and *Z*2 were chosen unevenly. In order to ensure comparability between the simulation and test results, the values of the system parameters were incorporated into the simulation model. The simulation was performed using *MATLAB Simulink*. The simulation and experimental results are illustrated in Figs. 6, 7, 8 and 9.

**Table 6:** System parameters.

Parameters	Value
C_1_, C_2_, C_3_, C_4_, C_5_, and C_6_	1000 uF electrolytic capacitor paralleled with 1.5 uF polyester capacitor
L_1_, L_2_, L_3_ and L_4_	1200 uH
Resistance of L_1_ and L_2_	0.01 Ω
Primary windings of transformers	44 turns
Secondary windings of transformers	22 turns
Leakage inductance of transformers primary winding	2 uH
Leakage inductance of transformers secondary winding	1 uH
Resistance of transformers primary winding	0.02 Ω
Resistance of transformers secondary winding	0.01 Ω
D_1_, D_2_, D_3_, and D_4_	U1560 from Thinki Semiconductor
Gate drive	ICL7667 from MAXIM
Optocoupler	6N137 from VISHAY
All power transistors	IRFP460 from VISHAY
${\text{D}}_{{\text{st}}{1}}$	0.29
${\text{D}}_{{\text{st}}{2}}$	0.20
Modulation index	0.80
${\text{V}}_{{\text{i}}{1}}$	30 Volt
${\text{V}}_{{\text{i}}{2}}$	30 Volt
Switching frequency Z1	15 kHz
Switching frequency Z2	4 kHz
Fundamental frequency	50 Hz
Load (per phase) (delta connection)	45 Ω
Controller of Z1 and Z2	PIC18F452 from Microchip
*Magnetic cores*	Ferit EE6565 from Magnetic

The average values for the currents absorbed from input sources 1 and 2 in simulation and test results are 4.6, 2.9, 4.7, and 2.6 Amperes, respectively, as shown in Figs. 6a,b, and 9a. Additionally, the voltage of the independent sources connected to the converters inputs are shown in Fig. 8a and b. As can be seen in these figures the proposed converter is capable of supplying the load with one or both of *V*_*i*1_ and *V*_*i*2_ Since there are portions of times in which one or both of *V*_*O*1_ or *V*_*O*2_ have non-zero voltages simultaneously. Also, Fig. 8d and the test results (Fig. 9c) show that the value of DC-link voltage (${\text{V}}_{{\text{O}}{2}}$) in the NSTS in simulation and test results to be 92 and 94 Volts, respectively. The transformer's primary winding current in the NSTS essentially needs to be equal to the current of the magnetizing inductors for the system function properly (*i*_*L*1_ and *i*_*L*2_). And since at this time, the magnetizing inductors of the transformers are discharged, their currents need to be reduced. Also, at the same time, the currents of the secondary windings have to be zero. With these explanations and by referring to Figs. 6f and h and 9b and f, it can be seen that the primary and secondary windings currents are in line with the expected outcomes. In the shoot-through time, however, both primary and secondary windings are conducting. The primary winding currents, minus their magnetizing currents, are multiplied by the turn ratio ($\text{n} = \frac{{\text{N}}_{1}}{{\text{N}}_{2}}$) and flow in the secondary windings, given that the turn ratio of the transformers in the proposed system is 2. In this regard, it is also observed that in Figs. 6f and h and 9b and f, in the shoot-through time, if the magnetizing current (which is approximately equal to the amount that flows through the primary windings in the NSTS) is subtracted from the primary windings current and then multiplied by 2 (turn ratio), the secondary windings current is obtained. In this regard, it is also noticed that, in terms of quantity, the curves of the transformers' windings currents in simulation studies and experiments with good accuracy are equal. As regards such other variables as inductors voltage and current, capacitors voltage, and so on, it can be seen that the simulation results (Figs. 6 and 7) verify the experimental results presented in Fig. 9. A few differences between the experimental and simulation results are due to the simulations using specific prototype parasitic elements and a lack of PCB modeling.

To calculate the efficiency of the proposed inverter in the fundamental frequency (50 Hz), the input and output active powers are to be computed. Figure 9a shows that the active power generated by the sources is:${\text{V}}_{{\text{i}}{1}}\times {\text{I}}_{{\text{i}}{1}}+{\text{V}}_{{\text{i}}{2}}\times {\text{I}}_{{\text{i}}{2}} = {30}\times \text{4.7}+{30}\times {2.6} = {219}$ watt; and Fig. 9h shows the active power absorbed by the load is:${3}\times {\text{R}}\times {\text{I}}_{\text{rms-phase-}{50}{\text{Hz}}}^{2} = {3}\times {45}\times ({\frac{1.7}{\sqrt{2}})}^{2} = {199.1}$ watt. Therefore, the efficiency of the system is determined at about 91%.

Some high-frequency noises, observable in Fig. 9h, are related to non-ideal and parasitic elements of the output filter. This causes some high-frequency current components to pass through the output filter.

For evaluating the contribution of the sources in supplying the load, the value of $\frac{{P}_{i1}}{{P}_{i2}}$ needs to be calculated. Figure 4 (b) shows if $\frac{{\text{V}}_{{\text{i}}{1}}}{{\text{V}}_{{\text{i}}{2}}} = {1}\text{; n=}\frac{1}{{2}}$, $\frac{{\text{P}}_{{\text{i}}{1}}}{{\text{P}}_{{\text{i}}{2}}}$ is equal to 1.6. If $\frac{{\text{P}}_{{\text{i}}{1}}}{{\text{P}}_{{\text{i}}{2}}}$ is calculated according to the experimental results (Fig. 9a), $\frac{{\text{P}}_{{\text{i}}{1}}}{{\text{P}}_{{\text{i}}{2}}}$ is equal to 1.8. Some differences between Figs. 4b and 9a in calculations of $\frac{{\text{P}}_{{\text{i}}{1}}}{{\text{P}}_{{\text{i}}{2}}}$ are related to the ideal system consideration in section “An analysis of steady state relations and power-sharing”.

Figures 7d–f, 8d and 9c,d show the leakage inductors of the transformers used in the suggested inverter do not cause voltage spikes on DC-link and line-line voltages. Therefore, using the inverter in high-power applications requires no such considerations as protection and control of the switches and their switching losses.

**Figure 10:**
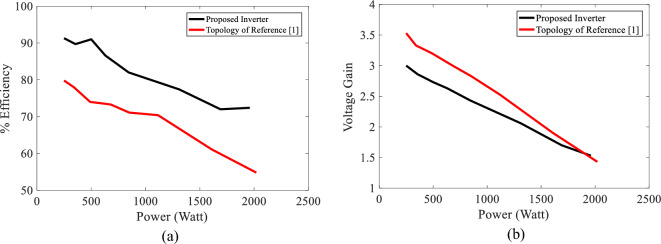
Comparing efficiency and voltage gain of the proposed inverter and that of topology of Ref.^1^ versus power while considering parasitic elements according to Table 6.

Figure 10a and b show that if parasitic elements of the system and the topology of Ref.^1^ are considered, the proposed inverter has higher efficiency (more than 10%) while the voltage gain of the proposed inverter at powers higher than 1700 watts is almost equal to that of the topology in Ref.^1^. Therefore, eliminating some active and passive components from the topology of Ref.^1^ brings about improvements in cost, weight, volume, and efficiency in high-power applications.

Efficiency is one of the most crucial factors in the performance evaluation of an inverter. A comparison was made between the proposed inverter and some of the references mentioned in the literature. The efficiencies of the proposed inverter and those in previous works have been shown in Table 7. In this comparison, it should be considered that the proposed inverter is a single-stage, high voltage gain, microcontroller-based inverter which takes advantage from machine intelligence in its protection procedure. Therefore, this inverter should be compared with other works that have similar features. The inverters presented in Refs.^9–11,32,34,35,38–42^ are all single-stage non-microcontroller-based inverters that have a low voltage gain. Also, these inverters don’t take advantage from machine intelligence in their structure. The inverter described in reference^37^ is a single-stage, microcontroller-based inverter with a low voltage gain that does not benefit from machine intelligence in its structure. The inverter described in Ref.^24^ is a single-stage, microcontroller-based inverter with a high voltage gain, but it does not incorporate machine intelligence into its design. At the end, only the inverter presented in Ref.^1^ has all the same features mentioned for the proposed inverter in this paper. Based on the efficiency values of Table 7 it can be concluded that the proposed inverter has a higher efficiency in addition to having a lower semiconductor count with respect to the inverter presented in Ref.^1^.

**Table 7:** Efficiency values for the proposed inverter and previous works.

Reference Num	% Efficiency
^32^	98.5
^10^	98.5
^9^	98.19
^27^	98.1
^33^	98
^35^	97.5
^37^	97.5
^11^	97.2
^4^	96.82
^12^	96.8
^28^	96.7
^29^	96
^18^	96
^34^	95.5
^38^	95.4
^39^	95.2
^5^	95
^41^	94.2
^25^	94
^6^	94
^7^	93
^24^	92
^8^	91
Proposed inverter	91
^40^	90.7
^42^	90.48
^30^	90
^31^	90
^36^	90
^1^	87.1
^26^	85

## Conclusion

The present research paper sets forth a multi-port three-phase inverter. This structure is based on single-stage conversion, and besides employing six switches of the classic single-input inverter, it only uses one extra switch. This structure suits such applications as hybrid renewable energy systems as it boosts voltage. Artificial intelligence based on hardware design is exploited in the proposed inverter, precluding the need for human intervention or circuit protection by DC-link electrolytic capacitors. Also presented in this paper are explanations related to the operation of the proposed inverter together with relations governing the system and the curves explaining power-sharing and voltage gains. The possibility to individually control the power ratio absorbed from the input sources and the DC-link voltage is demonstrated. The proposed inverter is further shown to exhibit higher efficiency in high-power applications. The operation of the prototype has been verified through simulation studies using prototype parameters.

## Data Availability

Data and codes for simulation-based items can be furnished on demand from the corresponding author.
