# Publisher Correction: A single-stage dual-source inverter using low-power components and microcomputers

**DOI:** 10.1038/s41598-024-54747-w

**Published:** 2024-02-20

**Authors:** Majid Ghani Varzaneh, Amirhossein Rajaei, Navid Kamali-Omidi, Ali Shams-Panah, Mohamadreza Khosravi

**Affiliations:** 1 Shiraz University of Technology, Shiraz, Iran; 2 Islamic Azad University of Dezful, Dezful, Khozestan Iran; 3 Islamic Azad University of Najafabad, Najafabad, Isfahan, Iran; 4 Weifang University of Science and Technology, Shandong, China

Correction to: *Scientific Reports* 10.1038/s41598-024-51513-w, published online 20 January 2024

In the original version of this Article Mohamadreza Khosravi was incorrectly affiliated with ‘Islamic Azad University of Najafabad, Najafabad, Isfahan, Iran’. Their correct affiliation is listed below.

Weifang University of Science and Technology, Shandong, China.

Additionally, Ali Shams-Panah was incorrectly affiliated with ‘Weifang University of Science and Technology, Shandong, China.’ Their correct affiliation is listed below.

Islamic Azad University of Najafabad, Najafabad, Isfahan, Iran.

The original Article has been corrected.

